# Caries Index and Salivary Factors in Children: A Case–Control Study

**DOI:** 10.3390/children12121631

**Published:** 2025-12-01

**Authors:** Clara Sandibel Garcete Delvalle, Judit Carrasco Vivó, Guillermo Reichard, Leyre Prado Simon, Marta Bruna del Cojo, Eva María Martínez Pérez, Sergio Portal Nuñez

**Affiliations:** 1 Department of Dentistry, School of Medicine, CEU San Pablo University, 28668 Madrid, Spainleyre.pradosimon@ceu.es (L.P.S.); marta.brunacojo@ceu.es (M.B.d.C.); evamaria.martinezperez@colaborador.ceu.es (E.M.M.P.); sergio.portalnunez@ceu.es (S.P.N.); 2 Department of Dental Clinical Specialties, School of Dentistry, Faculty of Dentistry, Complutense University of Madrid, 28040 Madrid, Spain

**Keywords:** dental caries, pediatric dentistry, oral health

## Abstract

Background: Dental caries is currently the most common chronic disease affecting the Spanish pediatric population. Therefore, the main objective of this study was to assess Caries Management by Risk Assessment (CAMBRA) in children attending a university dental clinic between the ages of 6 and 12 and establish the DMFT (decayed, missing, and filled teeth index for permanent teeth) of the sample. In addition, the study calculated the plaque index, salivary pH level, buffering capacity, and the quantity and quality of saliva in the sample and determined if there were statistical differences between sexes and between subgroups with DMFT = 0 (control) and DMFT > 0 (cases). Methods: A case–control study was conducted with 89 patients aged 6 to 12 years. Clinical and salivary indicators were measured. Caries risk was also assessed using the CAMBRA protocol, and the sample was also divided into control and case groups for further analysis. Results: The CAMBRA questionnaire showed that 65.2% of participants presented a high risk of caries. The sample showed an average DMFT of 0.65 with a plaque index of 57.2%. The mean salivary pH was 7.35 (±0.30). The average volume of stimulated saliva was 6.83 mL (±3.68), and the buffering capacity was classified as normal in 57% of cases. No statistically significant differences were found between sexes or in the evaluated risk factors. In the control group, the mean salivary pH value was 7.35, and the amount of stimulated saliva was 6.5 mL. The buffering capacity was classified as normal in 67% of cases. In contrast, the case group presented a mean pH value of 7.15 and a salivary volume of 5 mL, with a normal buffer capacity in 50% of cases. Conclusions: The CAMBRA protocol indicated that more than half of the participants presented a high risk of caries, indicating the need to customize treatment plans for each patient. The salivary pH showed statistic differences between the control and case groups, indicating the importance of incorporating salivary biomarkers into routine clinical practice.

## 1. Introduction

Dental caries is currently the most prevalent chronic disease in the Spanish pediatric population [1]. In a recently study conducted in 2022, seven out of ten children under six years old who attended the Policlinic University CEU San Pablo were affected by early-childhood caries [2]. However, this pathology is not exclusive to Spain but represents a global health problem [3]. According to the World Health Organization (WHO), caries affects between 60% and 90% of the pediatric population worldwide, mostly in communities with limited dental health services [4].

This disease is due to an imbalance between the demineralization and remineralization processes of tooth enamel, with the demineralization process prevailing [4]. Bacteria present in dental biofilm, especially S. Mutans, are the main etiological agents of caries [3,4,5]. However, these bacteria cannot cause cavities without the presence of a suitable substrate, which is why caries is considered a sugar-dependent disease [3]. In addition, oral biofilm is not only the main cause of caries but also the main cause of periodontal disease [6].

Dental caries is considered a multifactorial disease in which biological, behavioral, environmental, and even socioeconomic factors interact in parallel [4,7]. In addition, even gender, age, and geographic location have been linked to the development of dental caries [8]. Other risk factors to take into consideration include gingival inflammation and the consumption of sugary foods or fermentable carbohydrates between meals. In addition, inadequate saliva flow, certain medications that affect saliva, the presence of deep pits and fissures, and exposed roots, among other factors, contribute to the accumulation of bacterial plaque and increase the risk of caries [3]. Genetic variability affecting the composition of saliva can also alter its buffering capacity and increase the risk of caries [9]. In addition, enamel structure and actual dentin strength can be influenced by genetic factors linked to greater susceptibility to caries in certain population groups [3,10]. Another risk factor is the parents’ education level, as it strongly influences their family income, subsequent socioeconomic status, and access to dental care. Previous research has demonstrated that parents with lower educational and socioeconomic status tend to have children with a higher prevalence of caries [11,12,13]. In fact, according to Perinetti et al., many of the risk factors mentioned above, such as socioeconomic status, dietary habits, and oral hygiene, were present in their Italian OHSAR Survey conducted among schoolchildren [8].

On the other hand, among the protective factors is saliva itself as a defense mechanism against caries. Bacterial acidity is usually neutralized by salivary mechanisms in the oral cavity [4]. In addition, other key strategies for the prevention of caries include professional application of fluoride varnish, encouraging daily brushing with fluoride toothpaste, daily use of fluoride mouthwashes and dental flossing, and encouraging a diet low in cariogenic sugars. Also, biannual application of fluoride varnish, xylitol tablet consumption, monthly use of chlorhexidine, and the application of calcium and phosphate toothpastes have also been used to prevent the development of caries. In addition, community-based dental measures include water fluoridation and public education [14].

The global need to manage and reduce caries has led to various preventive protocols worldwide. Among these is the CAMBRA protocol (Caries Management by Risk Assessment) [7,15], originally designed to help prevent, treat, and control dental caries by taking into consideration the different risk and protective factors of caries [7]. One of the key features of the CAMBRA protocol is that it attempts to design an individualized treatment plan based on established factors [7]. According to the protocol, different risk levels are then established, these being classified as low, moderate, high, and extreme, delivering a specific recommended action plan for each case. This protocol has managed to significantly reduce the incidence of caries and promote oral health among pediatric patients since its establishment [16,17].

In addition, the plaque index is a useful, inexpensive, and easy-to-use tool for assessing a patient’s oral hygiene status and indicates the presence of specific bacteria in the biofilm [18]. Previous research has already confirmed that the presence of high levels of bacterial plaque in the plaque index leads to carious lesions [19].

### Aim of the Present Study and Hypothesis

Although the CAMBRA protocol is widely used, its effectiveness in the Spanish pediatric population and the relationship between salivary factors and caries risk in this context have not yet been fully evaluated. Due to this and the high incidence of caries in the Spanish pediatric population, this study was justified. The primary objective was to assess the CAMBRA caries risk in our sample of children between the ages of 6 and 12. This investigation involved determining the sample’s DMFT, calculating the plaque index, salivary pH level, and the quantity and quality of saliva in the sample, and determining whether there were differences between the sexes. Additionally, we aimed to calculate the saliva pH, its buffer capacity, and quantity in children with DMFT = 0 and those with DMFT greater than 0. Therefore, as a null hypothesis, we proposed that there are no differences in risk factors between children with DMFT = 0 and DMFT greater than 0, and that any differences are due to chance.

## 2. Method and Procedures

### 2.1. Study Design and Approval by Ethics Committee

This is an observational case–control study, where the cases are children with DMFT > 0 and the controls are subjects with DMFT = 0. The study was approved by the university’s research ethics committee (approval code 515/21/44). All guardians signed informed consent forms regarding the transfer of the data from their medical records, allowing their use for research purposes. All the data was collected during the first visit appointment.

### 2.2. Sample Selection

Subjects were selected from patients attending the university’s Master’s in Pediatric Dentistry program for a first visit during the following timeframe: October 2024 to March 2025. Participants were selected according to the following selection criteria: Any clinically healthy 6- to 12-year-old was included in the study. All subjects whose guardian refused to participate in the study, those who had ingested food, beverages, chewed gum, or any candy within 30 min prior to the test, and subjects who had brushed their teeth or rinsed their mouth with mouthwash within half an hour prior to the appointment were excluded. Additionally, any subject who had been receiving antibiotics or using antimicrobial mouthwashes in the past 7–14 days and children with motor or cognitive difficulties that prevented them from performing the test correctly were excluded. Subjects were selected consecutively as long as they met the aforementioned requirements and participated within the previously established limited timeframe. Due to the fact the participants had to adhere to the academic calendar and to the student’s schedule, no power calculation was performed prior to the study. In order to control and prevent confounding, subjects in the case group were paired with participants in the control group who had similar characteristics such as age and gender as we only included 6- to 12-year-old boys and girls.

### 2.3. Description of Data Collection and Parameters Studied

Prior to data collection, one of the main investigators gave a theoretical lecture to the collaborators to standardize the methodology and ensure accurate data measurement. The forms were then completed. During data collection, the researchers followed the caries risk assessment CAMBRA form proposed by Featherstone et al. [15]. Risk factors taken into consideration included the following: visible plaque, snack consumption, inadequate saliva, reducing factors, medications, deep pits and fissures, exposed roots, orthodontic treatments, prosthesis, and defective treatments. Protective factors taken into account were the following: availability of fluoridated water, fluoride toothpaste, fluoride mouthwashes, complementary hygiene methods, fluoride varnish, xylitol chewing gum, monthly use of chlorhexidine, calcium and phosphate toothpastes, and adequate saliva. After gathering the information, the CAMBRA questionnaire allocates each patient under one of the following caries risks:

1. Low caries risk:
  • No incipient carious lesions, no active caries, or restorations in the last three years. No risk factors, so protective factors predominate.
2. Moderate caries risk:
  • One or two incipient caries lesions, active caries, or restorations in the last 3 years. More risk factors than low-risk patients, exceeding the protective factors.
3. High caries risk:
  • Three or more incipient lesions, active caries, or restorations over the last 3 years. Multiple risk factors present.
4. Extreme caries risk:
  • High-risk patients, in addition to salivary hypofunction.

To ensure anonymity, all questions that the children’s parents or legal guardians were asked were recorded using only the medical record number, without including names or any identifying information.

### 2.4. Saliva-Related Parameter Assessment

After completing the data collection forms, salivary parameters and the plaque index were measured. To assess salivary flow, each participant was given a piece of paraffin gum and a measuring cup. The child was given five minutes to fill the cup with as much saliva as possible. The collaborator then recorded on the form the amount of saliva collected, extracting it with a pipette. Salivary flow was classified as follows: normal flow: above 5 mL; low flow: 3.5–5 mL; and very low flow: any sample under 3.5 mL.

Subsequently, three drops of the collected saliva were placed on the saliva buffer strip and left to react for two minutes to evaluate quality of the saliva. After this time, the color obtained on the strip was observed and recorded according to the following scale provided by the manufacturer: green: 4 points; green/blue: 3 points; blue: 2 points; blue/red: 1 point; and finally, red: 0 points. Normal buffering capacity was defined as any score between 10 and 12, while low buffering capacity was defined as a score between 6 and 9, and very low as a score under 6.

Finally, saliva pH was assessed using GC Dental Care^®^ brand test strips following the manufacturer’s instructions. To do this, the strip was inserted halfway into the saliva sample and then removed. Ten seconds was allowed to pass for a color change to take place, and the strip was then compared to the kit’s reference table. The saliva pH was then recorded on the data collection sheet.

### 2.5. Plaque Index

The plaque index was assessed using the Tri Plaque ID Gel^®^ plaque revealer, manufactured by GC Dental Care^®^ (Lucerne, Switzerland). The plaque revealer was applied on top of the tooth surface, staining the accumulated biofilm. The plaque presence on each tooth was then recorded using the O’Leary index, considering the plaque located at the gingival margin and dividing each tooth into four zones. Finally, the percentage of surfaces with plaque was calculated using the following formula: number of stained surfaces divided by the total number of surfaces and multiplied by 100.

### 2.6. dmft Index

The dmft index (decayed, missing, and filled teeth index for primary teeth) was calculated by summing the number of decayed teeth, missing teeth (when the reason for their absence was caries), and filled teeth in deciduous dentition for each individual. This total was divided by the number of individuals studied to obtain the dmft index for the entire study population. Crowns were counted as fillings if their presence was due to caries, and as healthy teeth if their presence was due to another cause, such as trauma. If a tooth had both a filling and caries, it was considered solely carious [20].

### 2.7. DMFT Index

The DMFT index (decayed, missing, and filled teeth index for permanent teeth) was determined by summing the number of carious first permanent molars, missing molars (if the reason for their absence was caries), and filled molars for each case, obtaining a score between zero and four points. The total number of these molars was divided by the number of individuals studied to obtain the DMFT for the study population. As with the dmft index, in cases of patients with dental crowns, these were recorded as fillings if the reason for their presence was caries and as healthy teeth if their presence was due to another reason. If a filling and caries were present simultaneously, the tooth was considered solely carious [21].

All the data was collected using caries risk forms, which was subsequently tabulated in Microsoft Excel^®^ for further analysis.

However, the variables mentioned above can also be described as outcomes, exposures, predictors, potential confounders, and effect modifiers. In our study, the outcome will be the risk of caries, while the exposure is the factor being studied (different risk factors and protective factors described in the CAMBRA questionnaire, in addition to the DMFT and dmft indexes, and the saliva variables). Predictor variables that might influence the outcome include socioeconomic status and geographical area as this is an urban setting. A potential confounder is a variable that can distort the relationship between an exposure and an outcome, such as age, because older children have been exposed to the risk factors for a longer period. An example of an effect modifier would be gender, as it can modify how the risk of caries (the outcome) differs between boys and girls in our study.

### 2.8. Statistics

The study was divided into two statistical phases: the first was a descriptive analysis and the second an inferential analysis, with the aim of exploring the distribution of the study variables and the associations between risk factors related to dental caries. All statistical analysis was carried out with R-Studio version 4.2.1© software.

For the general quantitative variables, the mean, minimum and maximum values, and standard deviation were calculated. DMFT and dmft scores for the affected individuals were also calculated, that is, for those participants with caries and a score other than zero.

All these variables mentioned above were calculated for the general sample and then according to subgroups classified as DMFT = 0 and DMFT > 0. They were also calculated according to gender to assess whether there were differences between males and females.

For dichotomous categorical variables, the percentage of affirmative or negative responses to the different variables was calculated for the overall sample and according to subgroups with DMFT = 0 and DMFT > 0. For ordinal categorical variables, the sample distribution was calculated for the general sample and according to subgroups.

In this phase, the chi-square analysis was used for categorical variables. The Shapiro–Wilk normality test was also applied to assess the assumption of normality of the variables, considering those with a *p* value > 0.05 as normal. Student’s *T*-test was carried out for those variables where normality could be assumed, while the Mann–Whitney U test was used for the study of those variables where normality could not be assumed, both with a 95% confidence interval. We decided prior to the study that if there was any missing research data, it would be addressed by removing incomplete cases.

## 3. Results

**Figure 1:**
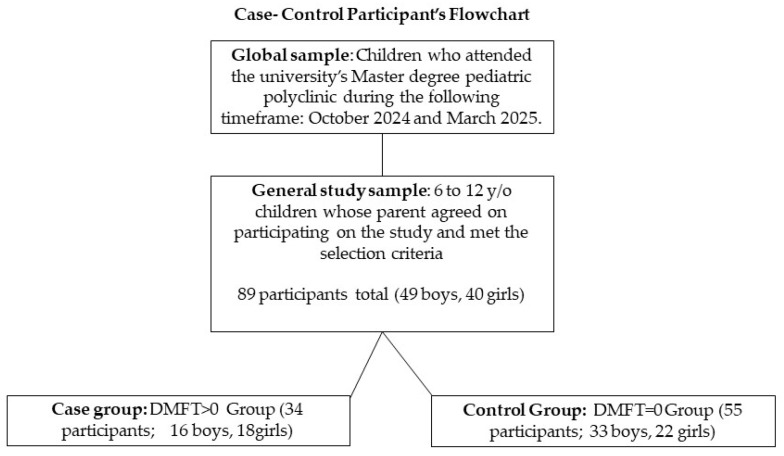
Participant flowchart diagram.

The descriptive variables reflected the sample, which comprised 89 pediatric patients, with a distribution of 49 (55.1%) boys and 40 (44.9%) girls. When the general sample was subgrouped, the study group represented 61.7% of the total sample, while the control group represented 38.2% (Figure 1). All 89 children from the general sample participated. There is no missing data. The mean age was 8.24 years, with a relatively symmetrical distribution of 8.46 for females and 8.05 for males. The oldest recorded age was 11.98 years, and the youngest age was 6.07 years. The dmft index presented an overall mean score of 4.29, with a maximum value of 20 and a minimum of 0. Furthermore, the caries frequency, or percentage of individuals with at least one primary tooth affected by the disease, was 80.9%.

On the other hand, the DMFT index showed an overall mean score of 0.65, affecting 38.2% of the teeth. The amount of stimulated saliva had an overall mean value of 6.83 mL (±3.68), with a maximum value of 20 mL and a minimum value of 1 mL. Furthermore, the salivary pH recorded an overall mean value of 7.35 (±0.30), with a maximum value of 8 and a minimum of 6.6. After dividing the descriptive statistics of the quantitative variables according to the DMFT = 0 and DMFT > 0 groups, the results were recorded in Table 1.

### 3.1. Caries Risk

According to the data, 65.2% of the sample (62.5% of girls and 67.3% of boys) were at high risk for caries, followed by the medium-risk group, which represented 21.3% of the overall sample (25% of girls and 18.4% of boys). However, only 13.5% of the overall sample (12.5% of girls and 14.3% of boys) fell into the low-risk category. No patients were classified as extreme-risk, and no significant differences were observed between genders.

### 3.2. Salivary Factors and Sample Distribution by Buffer Capacity

The mean overall stimulated saliva volume was 6.83 mL over a five-minute period, equivalent to 1.36 mL/min. The mean by gender was 5.99 mL (1.19 mL/min) in females and 7.5 mL (1.5 mL/min) in males. A mean salivary pH of 7.35 was observed, being 7.32 in females and 7.38 in males. Finally, the buffer capacity was classified as normal in 57% of the subjects, low in 33%, and very low in the remaining 10%. In the female population, the buffer capacity ranked as normal in 50% of cases, low in 40% of cases, and very low in 10% of cases. However, the buffer capacity classification in the case of males was 63%, 27%, and 10%, respectively. In the control group, buffering capacity was classified as normal in 62% of cases, low in 27% of cases, and very low in the remaining 11%. However, in the DMFT > 0 group, buffering capacity was classified as normal in 50% of cases, low in 41% of cases, and very low in the other 9% of the sample.

### 3.3. CAMBRA Questionnaire

Regarding parental responses to the CAMBRA questionnaire, the three most common risk factors in the general population were the presence of visible plaque (61%), frequent snacking (44%), and the presence of deep pits and fissures (30%), as shown in Table 2. The most common protective factors in the total sample were adequate saliva (78%), brushing with fluoride toothpaste at least once a day (79%), and the application of fluoride varnish in the last 6 months by a dentist (26%), as shown in Table 3.

When we evaluated these responses based on the sample’s DMFT, after arranging the sample by DMFT = 0 and DMFT > 0, the percentages varied, but the order of frequency did not change. The only exception was the in the DMFT > 0 group, where the third most common risk factor was inadequate saliva, with the same percentage as snacking.

Regarding age, the Shapiro–Wilk test indicated that normality could be assumed in both groups to be *p* = 0.20 for the DMFT = 0 control group and *p* = 0.15 for the DMFT > 0 case group. Student’s *t*-test (*p* value = 0.04) indicated that age was a significant variable.

**Figure 2:**
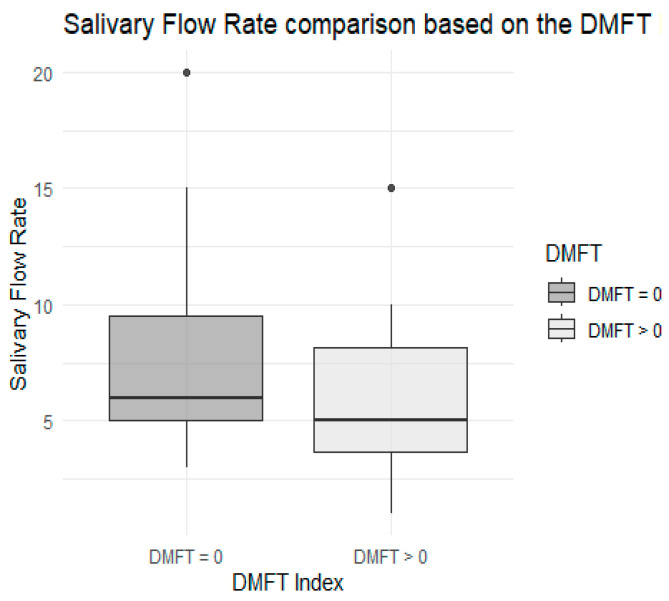
Comparative boxplot regarding saliva quantity, by case–control subgroup.

Regarding the amount of stimulated saliva, after performing the Shapiro–Wilk test, it was not possible to assume normality in either group because the *p* value was 0 for the DMFT = 0 group and *p* = 0.008 for the DMFT > 0 group. On the other hand, the Mann–Whitney U test (*p* = 0.06), although close to the significance threshold, did not show significant differences among the two groups either, as described in Figure 2.

**Figure 3:**
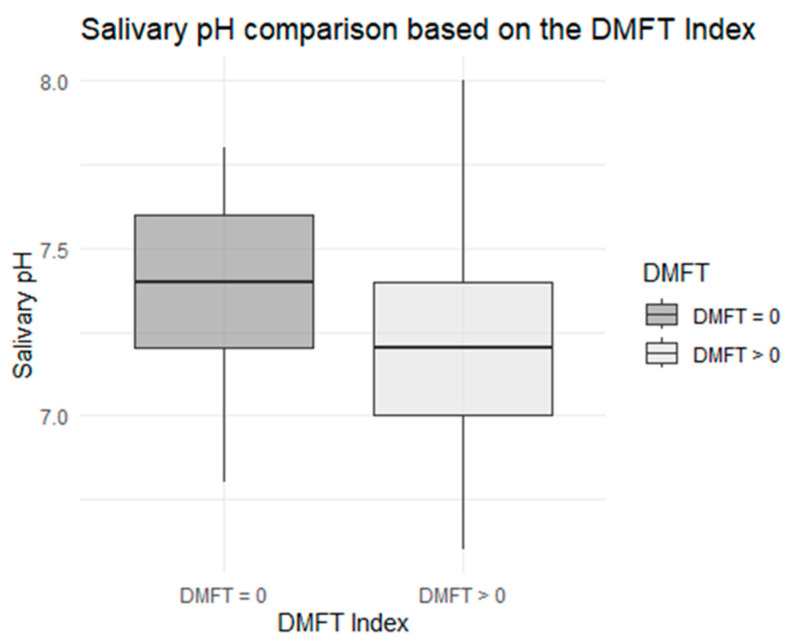
Comparative boxplot regarding salivary pH, by case–control subgroup.

Regarding salivary pH, the Shapiro–Wilk test did not reflect a normal distribution for the DMFT = 0 group (*p* = 0.0). However, the test for normality showed normal assessment for the DMFT > 0 group (*p* = 0.145). The Mann–Whitney U test was performed because there was no normal distribution for both groups. The Mann–Whitney U test obtained significant differences between both groups (*p* = 0.043), as described in Figure 3.

Lastly, according to the results of the chi-square test, no significant relationship between buffer capacity and DMFT could be confirmed (*p* = 0.39). Regarding the visible plaque, the chi-square test also failed to confirm a significant relationship between visible plaque and DMFT (*p* = 0.95).

Therefore, based on the results above, where there are differences in risk of caries between children with DMFT = 0 and DMFT greater than 0, we reject the null hypothesis.

## 4. Discussion

This research indicates a high prevalence of caries in primary dentition (81%). Although the DMFT index was low (0.65), the involvement of approximately 38% of patients with at least one decayed first permanent molar is concerning because it indicates the onset of caries at an early age as these are young children. This finding is consistent with similar research conducted in high-risk samples in Latin America [22,23], where caries in the mixed dentition occurs at an early age. However, another study in a Spanish population showed lower prevalence, perhaps because these subjects had greater access to preventive treatments than those in our specific community [24].

The CAMBRA questionnaire showed that more than half of the participants (65.2%) presented a high risk of caries, indicating the need to customize treatment plans for each patient. Historically, this questionnaire has achieved sufficient sensitivity to detect patients with accumulated risk factors, so we believe it is a useful tool for planning preventive dental treatments [3]. However, according to our sample results, most of our children did not present dental cavities during the first visit. This contrasts with the CAMBRA results as it takes into account caries that were resolved during the last three months prior to the screening; therefore, many high-risk patients according to the questionnaire were actually caries-free during our assessment.

According to the tests performed, during the inferential analysis, age was an influential factor (*p* = 0.04). Therefore, age is a variable that plays an important role in the presence of caries in the study sample. The older the age, the higher the DMFT index. Although it is true that the older the molars, the longer the exposure to risk factors, this indicates that better programs focused on effective preventive treatments are still needed. According to the longitudinal study conducted by Winter et al., caries progresses with age and in the absence of intervention [20].

On the other hand, regarding the amount of stimulated saliva, although no statistically significant difference was found between the two study groups (*p* = 0.06), the fact that the value was close to the threshold of significance leads us to recommend that this be investigated with a larger sample size in the future. Equally important is that the DMFT > 0 group had a lower mean stimulated saliva volume (6 mL) than the DMFT = 0 group (7.33 mL), indicating that the case group has less protection against caries from salivary flow than the control group. This pattern is similar to previous studies [25,26], which indicate that decreased salivary flow is associated with a risk factor that increases the likelihood of caries.

Furthermore, the inferential analysis observed a significant difference (*p* = 0.043) in salivary pH levels, with the saliva pH being more alkaline in the control group and more acidic in the case group. This implies that salivary pH is significantly related to the development of caries in subjects with more acidic salivary pH. This underscores the importance of salivary pH as a clinical indicator and risk factor, since the literature demonstrates that a low pH promotes demineralization, and inadequate buffering capacity increases the risk of caries. This finding is consistent with current evidence and reinforces the inclusion of salivary pH as a routine parameter in individualized management protocols [3].

In addition, no significant differences were found regarding saliva buffering capacity (*p* = 0.39), perhaps due to limitations in the sample size or the effect of other compensating factors, such as hygiene or diet. However, the predisposition to a low buffering capacity of 41% in the case group compared to 27% in the control group indicates that this variable should always be considered in epidemiological studies of dental caries.

Additionally, analysis of the CAMBRA questionnaire showed that the three most prevalent risk factors were the presence of visible plaque, frequent snack consumption, and the presence of deep pits and fissures. Surprisingly, visible plaque did not show a significant relationship with DMFT (*p* = 0.95), a finding that contrasts with expectations and with other studies such as those conducted by Ramalho de Camargo et al., Morales et al., and Carvalho et al., in which there is a clear association between visible biofilm and the presence of active caries [5,18,24]. This could be due to possible underestimation during visual assessment, or possible remineralization mediated by protective factors.

However, according to the CAMBRA questionnaire, the most common protective factor was the use of fluoride toothpaste (79%), reflecting good hygiene practices in the sample. However, the use of mouthwashes, professional fluoride varnish, and complementary methods was much less frequent (<30%). The literature demonstrates the usefulness of these products in preventing caries, and therefore it is recommended to reinforce them through educational events aimed at prevention [3]. As mentioned before, an important protective factor is the amount of saliva, and a lower percentage of children in the DMFT > 0 group (68%) with “adequate saliva” compared to those in the control group (84%) confirms the importance of this parameter.

Therefore, not all factors assessed in the CAMBRA questionnaire were significant in the comparison of both study groups. Even so, these factors should not be ruled out, and future research with larger samples and long-term follow-up should be conducted to draw more precise conclusions. The distribution of risk and protective factors is consistent with that reported in the literature [25,27]. However, some key variables such as the presence of dental plaque or buffering capacity did not show a significant relationship with DMFT in this study, which could be influenced by the sample size or biases in data collection. Several studies have applied the CAMBRA protocol in pediatric populations [27,28], with samples of young children ranging from two years of age to adolescents, finding that its application can successfully reduce the incidence of caries in longitudinal follow-ups [16,28]; therefore, we also recommend its application in more mature adolescent populations.

Considering the results obtained and based on the scientific evidence examined, the null hypothesis proposed in this study can be rejected. Significant differences were found in the parameters analyzed (such as salivary pH, age, and saliva quantity) depending on the DMFT, supporting the existence of an association between these factors and the presence of the disease. These findings reinforce the validity of the indicators selected to estimate caries risk and justify the need for further studies that allow for better identification of vulnerable groups and the role played by each of the variables.

Finally, based on the results above, where there are differences in the risk of caries between children in the case and control groups, we rejected the null hypothesis. This is probably since many of our study variables, such as the salivary parameters, have been previously linked to caries.

### Limitations and Strengths

This research, although it provides valuable data on the implementation of the CAMBRA protocol in a pediatric sample from the Community of Madrid, also has limitations that must be considered. One of these is the retrospective, case–control study design. Therefore, this design cannot establish direct causality but rather an association between the case and control groups. Furthermore, it must be taken into account that dental caries is a multifactorial pathology, in which multiple factors simultaneously intervene. Another important limitation is the sample size, as it was limited to an established timeframe around the academic calendar. We thus recommend increasing the sample size in future research. Data collection through questionnaires reported by legal guardians represents another potential source of bias, as some questions, such as home hygiene habits, depend on accurate parental reporting. Another factor to consider is that this study was unable to collect socioeconomic data from the children’s environment, a factor that has been found to influence general health and prevalence of caries in children [13,21,23].

However, this study has strengths. From a clinical perspective, the implementation of the CAMBRA protocol is confirmed as a useful and viable tool for individualizing patient management based on their risk profile. The ability to classify children into risk categories, from low to high, allows pediatric dentists to design preventive or therapeutic measures tailored to each case. The fact that variables such as salivary pH show significant differences between patients with and without permanent molar involvement demonstrates the importance of incorporating salivary biomarkers into dental practice. Routinely measuring salivary pH or flow during dental checkups could help prevent the development or progression of caries [29]. In addition, these measures are relatively easy to manage, inexpensive, noninvasive, and reliable, so implementing them in dental practice should be straightforward.

From a health and public policy perspective, our results reinforce the need to strengthen preventive programs, primarily diet, mechanical plaque removal with a toothbrush, and the use of fluoride-containing toothpastes and mouthwashes [30]. Although a large proportion of participants reported using fluoride toothpaste, this was not the case with complementary hygiene methods such as mouthwashes and dental varnishes, creating a gap between clinical recommendations and their real-life implementation. This problem could potentially be rectified through educational campaigns at schools and health centers, educating both children and their parents [30].

This study provides a comprehensive view of the pediatric population attending university clinics, which can serve as a basis for developing standardized clinical protocols that integrate risk assessment tools such as CAMBRA at university institutions. In fact, this tool has been implemented by the university as all the patients who participated in this study received comprehensive and personalized dental treatment at the CEU San Pablo University’s polyclinic tailored to their caries risk and needs. Furthermore, this research could guide the design of future research seeking to evaluate the effectiveness of specific interventions based on risk levels, as well as longitudinal studies that aim to assess the progression of caries from early development.

Most of the patients who attend the university dental clinic come from low- to middle-income families. This is important to point out as the results will mostly reflect children living under this socioeconomic status. This means the results, although reflecting many children living in the Madrid community, cannot be generalized for the entire population.

The same applies to geography, as it plays a key role in caries prevalence, and our sample mainly represents children living a major city/urban area with more access to dental care than rural areas in Spain. In fact, in a study comparing Italian children living in urban and rural areas, caries indexes were significantly higher in schoolchildren living in rural towns than those living in main cities, and these disparities were even more marked as the children grew older [31]. To better assess the oral health in rural areas in Spain, more research will be required with children living in small rural towns, even if the sample size is reduced.

## 5. Conclusions

The CAMBRA protocol indicated that more than half of the participants presented a high risk of caries, indicating the need to customize treatment plans for each patient. The salivary pH showed statistic differences between the control and the case groups, indicating the importance of incorporating salivary biomarkers into routine clinical practice.

## Figures and Tables

**Table 1:** Descriptive statistics for case and control subgroups regarding quantitative variables.

	Mean		Standard Deviation		Minimum Value		Maximum Value	
	Control Group	Case Group	Control Group	Case Group	Control Group	Case Group	Control Group	Case Group
Age (years)	8.18	8.90	±1.56	±1.62	6.07	6.18	11.89	11.80
Amount of stimulated saliva (mL)	7.33	6	±3.78	±3.42	3.00	1.00	20.00	15.00
Salivary pH	7.4	7.27	±0.29	±0.30	6.80	6.60	7.80	8.00
dmft	4.19	4.45	±4.26	±3.15	0	0	20	11

Control Group: DMFT = 0 group; Case Group: DMFT > 0 group.

**Table 2:** Distribution of risk factors considered in the CAMBRA questionnaire in the general population and in the case and control groups.

List of Risk Factors	General Sample (%)	Control Group (%)	Case Group (%)
Visible plaque	61% (N = 54)	59% (N = 32)	62% (N = 22)
Snack consumption	44% (N = 39)	51% (N = 28)	32% (N = 11)
Inadequate saliva	22% (N = 20)	16% (N = 9)	32% (N = 11)
Reducing factors	7% (N = 7)	9% (N = 5)	7% (N = 2)
Medications	0% (N = 0)	0% (N = 0)	0% (N = 0)
Deep pits and fissures	30% (N = 27)	22% (N = 12)	44% (N = 15)
Exposed roots	3% (N = 3)	3% (N = 1)	4% (N = 2)
Orthodontic treatments	17% (N = 15)	15% (N = 8)	21% (N = 7)
Prosthesis	0% (N = 0)	0% (N = 0)	0% (N = 0)
Defective treatments	9% (N = 8)	9% (N = 5)	9% (N = 3)

Control Group: DMFT = 0 group; Case Group: DMFT > 0 group.

**Table 3:** Distribution of protective factors considered in the CAMBRA questionnaire in the general population and in the case and control groups.

List of Protective Factors	General Sample (%)	Control Group (%)	Case Group (%)
Availability of fluoridated water	11% (N = 10)	9% (N = 5)	15% (N = 5)
Fluoride toothpaste	79% (N = 70)	73% (N = 40)	88% (N = 30)
Fluoride mouthwashes	24% (N = 21)	22% (N = 12)	26% (N = 9)
Complementary hygiene methods	9% (N = 9)	12% (N = 7)	7% (N = 2)
Fluoride varnish	26% (N = 23)	24% (N = 13)	29% (N = 10)
Xylitol chewing gum	3% (N = 3)	4% (N = 2)	3% (N = 1)
Monthly use of chlorhexidine	3% (N = 3)	2% (N = 1)	6% (N = 2)
Calcium and phosphate toothpastes	8% (N = 7)	9% (N = 5)	6% (N = 2)
Adequate saliva	78% (N = 69)	84% (N = 46)	68% (N = 23)

Control Group: DMFT = 0 group; Case Group: DMFT > 0 group.

## Data Availability

The original contributions presented in this study are included in the article. Further inquiries can be directed to the corresponding author.
